# A plague on five of your houses - statistical re-assessment of three pneumonic plague outbreaks that occurred in Suffolk, England, between 1906 and 1918

**DOI:** 10.1186/1742-4682-7-39

**Published:** 2010-10-25

**Authors:** Joseph R Egan

**Affiliations:** 1 Microbial Risk Assessment, Emergency Response Department, Health Protection Agency, Porton Down, Salisbury, Wiltshire, SP4 0JG, UK

## Abstract

**Background:**

Plague is a re-emerging disease and its pneumonic form is a high priority bio-terrorist threat. Epidemiologists have previously analysed historical outbreaks of pneumonic plague to better understand the dynamics of infection, transmission and control. This study examines 3 relatively unknown outbreaks of pneumonic plague that occurred in Suffolk, England, during the first 2 decades of the twentieth century.

**Methods:**

The Kolmogorov-Smirnov statistical test is used to compare the symptomatic period and the length of time between successive cases (i.e. the serial interval) with previously reported values. Consideration is also given to the case fatality ratio, the average number of secondary cases resulting from each primary case in the observed minor outbreaks (termed *R*_*minor*_), and the proportion of individuals living within an affected household that succumb to pneumonic plague via the index case (i.e. the household secondary attack rate (SAR)).

**Results:**

2 of the 14 cases survived giving a case fatality ratio of 86% (95% confidence interval (CI) = {57%, 98%}). For the 12 fatal cases, the average symptomatic period was 3.3 days (standard deviation (SD) = 1.2 days) and, for the 11 non index cases, the average serial interval was 5.8 days (SD = 2.0 days). *R*_*minor *_was calculated to be 0.9 (SD = 1.0) and, in 2 households, the SAR was approximately 14% (95% CI = {0%, 58%}) and 20% (95% CI = {1%, 72%}), respectively.

**Conclusions:**

The symptomatic period was approximately 1 day longer on average than in an earlier study but the serial interval was in close agreement with 2 previously reported values. 2 of the 3 outbreaks ended without explicit public health interventions; however, non-professional caregivers were particularly vulnerable - an important public health consideration for any future outbreak of pneumonic plague.

## Background

Pneumonic plague is a disease that poses a threat to both civilian and military populations either via a biological aerosolised release or through zoonotic transmission [1]. Such routes of infection are not mutually exclusive since a biological attack in a non-endemic plague region could lead to reservoirs of plague-infected animals after the initial human infections have been controlled [2]. In addition, military populations are at risk when operating in plague endemic regions and the possibility of importation of plague from abroad also provides a continuing threat to public health in the U.K., and elsewhere [3]. It is therefore important to understand the epidemiology of pneumonic plague in order to mitigate any outbreaks of the disease. The Japanese are believed to have dropped plague-infected fleas over China during World War 2 [4] but due to a lack of detailed descriptions of biological attacks, researchers have previously analysed natural outbreaks to gain a better understanding of disease features such as the incubation/infectious periods and the potential for human-to-human transmission [3,5,6].

Prior to a single laboratory-acquired case of pneumonic plague at Porton Down in 1962,[7] the most recent English outbreaks occurred between 1906 and 1918 in Suffolk [8,9]. 3 outbreaks of pneumonic plague and 2 outbreaks of bubonic plague were believed to have resulted from shipping on the Rivers Orwell and Stour. The most likely explanation for these outbreaks is that grain brought from ports in the Black Sea and the Americas contained plague-infected rats which lead to enzootic rat-flea plague cycles. All of these outbreaks are particularly well documented and have been described as "unique to western Europe" [8]. Although they have been reported in previous papers, this study uniquely analyses the statistical epidemiology of the 3 pneumonic plague outbreaks. Unlike recent analyses,[10-12] the natural history and transmissibility of the Suffolk cases were unaffected by effective treatment since antibiotics were not available until ~30 years after the last Suffolk outbreak.

## Methods

**Figure 1:**
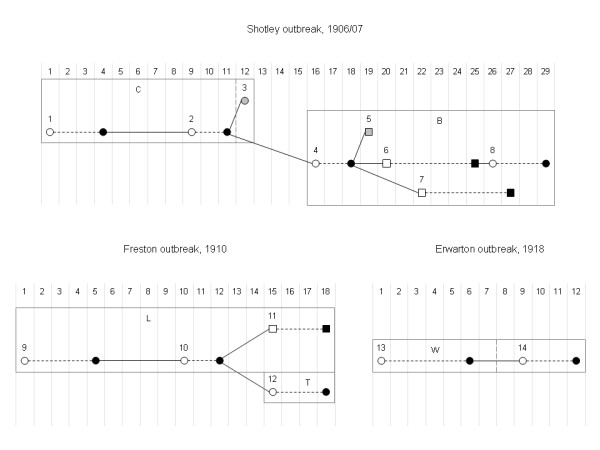
**Epidemic trees of the 3 pneumonic plague outbreaks**. The vertical grey lines separate the numbered days of each outbreak. Circles and squares represent female and male cases, respectively. White and black symbols represent time of symptom onset and death, respectively. Grey symbols represent time of symptom onset for those cases that recovered. Case numbers are given above time of symptom onset symbols. Dashed connectors represent the symptomatic period and un-dashed connectors represent routes of transmission. Boxes represent different locations and dividing long-dashed lines represent different cottages. C, B, L, T and W represent Charity Farm Cottages, Brickhill Terrace Cottages, Latimer Cottages, Turkey Farm Cottages and Warren Lane Cottages, respectively.

Table 1 provides data describing the 3 pneumonic plague outbreaks [9,13] and Figure 1 shows a graphical representation of the data using epidemic trees [10]. A brief explanation of each outbreak is given below.

**Table 1:** Outbreak data.

Case Number	Name	Age	Date of symptom onset	Date of death	Location	Symptomatic period (days)	Serial interval (days)	Number of secondary cases
Shotley, 1906/07								

1	Mrs C	53	9^th ^Dec.	12^th ^Dec.	Charity Farm Cottages	3	Index case	1

2	Mrs R	24	17^th ^Dec.	19^th ^Dec.	Charity Farm Cottages	2	8	2

3	Miss E. C	19	20^th ^Dec.	Recovered	Charity Farm Cottages	Recovered	3	0

4	Mrs G	46	24^th ^Dec.	26^th ^Dec.	Brickhill Terrace Cottages	2	7	3

5	Mr H. G	?	27^th ^Dec.	Recovered	Brickhill Terrace Cottages	Recovered	3	0

6	Mr G	56	28^th ^Dec.	2^nd ^Jan.	Brickhill Terrace Cottages	5	4	1

7	Mr R. G	7	30^th ^Dec.	4^th ^Jan.	Brickhill Terrace Cottages	5	6	0

8	Mrs W	66	3^rd ^Jan.	6^th ^Jan.	Brickhill Terrace Cottages	3	6	0

Freston, 1910								

9	Miss A. G	9	12^th ^Sept.	16^th ^Sept.	Latimer Cottages	4	Index case	1

10	Mrs C	40	21^st ^Sept.	23^rd ^Sept.	Latimer Cottages	2	9	2

11	Mr C	57	26^th ^Sept.	29^th ^Sept.	Latimer Cottages	3	5	Isolated

12	Mrs P	43	26^th ^Sept.	29^th ^Sept.	Turkey Farm Cottages	3	5	Isolated

Erwarton, 1918								

13	Mrs B	52	8^th ^June	13^th ^June	Warren Lane Cottages	5	Index case	1

14	Mrs G	42	16^th ^June	19^th ^June	Warren Lane Cottages	3	8	0

Columns 2 - 6 copyright The Trustee, The Wellcome Trust, reproduced with permission; originally published in [9].

### Shotley outbreak, 1906/07

The index case, Mrs C (case 1), who lived in Charity Farm Cottages, developed what is believed to be pneumonic plague on 9^th ^December 1906 and died 3 days later. She was nursed by her daughter, Mrs R (case 2), who subsequently developed the disease on 17^th ^December and died on the 19^th ^December. Given the close contact of the 2 cases it seems very likely that Mrs R was infected by her mother. Also, since evidence suggests that transmission takes place when cases are coughing bloody sputum and near death [14] then the approximate 5 day incubation period agrees with previously reported values [3,15]. Interestingly, another daughter, Miss C (case 3) also became ill on 20^th ^December but finally recovered. Miss C nursed both her mother and her sister; it was assumed that Miss C was infected by her sister given that the time-course of disease suggests she was less likely to have been infected by her mother.

The 2 daughters were both nursed by Mrs G (case 4) who lived approximately half a mile away at Brickhill Terrace Cottages. Mrs G became ill on Christmas Eve and died on Boxing Day; it was assumed that Mrs G was infected by Mrs R, the more seriously ill of the 2 daughters. Mrs G seems to have infected her husband (case 6) and 2 sons (cases 5 and 7) who all became symptomatic in quick succession between 27^th ^and 30^th ^December. The first son that experienced symptoms recovered. Mrs G's mother, Mrs W (case 8), travelled over 20 miles to attend her daughter's funeral and then remained at Brickhill Terrace Cottages to nurse her son-in-law and 2 grandsons. Mrs W became ill on 3^rd ^January 1907 and died 3 days later; it was assumed that infection occurred via Mrs W's son-in-law, Mr G, since he was the only case to have died (and thus experienced the late infectious stage) after Mrs W had arrived but prior to her onset of symptoms.

### Freston outbreak, 1910

Mrs C lived in Latimer Cottages with her husband, Mr C, and her 4 children from a previous marriage. On 12^th ^September 1910, Mrs C's daughter, Miss G (case 9), suffered a bout of vomiting and died 4 days later after having experienced a severe cough and diarrhoea. 5 days after the death of her daughter, Mrs C (case 10) began to experience similar symptoms and died after 2 days illness. 3 days after his wife's death, Mr C (case 11) and Mrs P (case 12), a neighbour living at Turkey Farm Cottages who had nursed Mrs C, also became ill. The following day local doctors isolated both cases in their homes in view of the infectious nature of the illness; other family members were requested to sleep in outhouses temporarily [16]. Mr C and Mrs P died on 29^th ^September; the same day that bacilli grown from blood specimens taken from these third generation cases were identified as *Yersinia pestis *(the causative agent of plague). Subsequently contacts of all cases were moved into isolation accommodation on 1^st ^October. The routes of transmission in this outbreak were relatively straight-forward to deduce; the only debatable link is whether Mr C was infected via his step-daughter or his wife. However, based on previous analysis [3,15] it is far more likely that Mr C experienced an approximate 3 day incubation period having been infected by his wife than incubating the disease for approximately 10 days after contact with the index case.

### Erwarton outbreak, 1918

Mrs B (case 13), who lived in Warren Lane Cottages, developed pneumonic plague symptoms on 8^th ^June 1918 and died 5 days later. Mrs B was visited by her next-door neighbour, Mrs G (case 14), who became ill on 16^th ^June. 2 days later the local general practitioner, Dr Carey (who had attended all cases in the Shotley and Freston outbreaks) visited Mrs G and suspected pneumonic plague after he found her with a high temperature, spitting blood and breathing rapidly. Mrs G died the following day at approximately the same time that pneumonic plague was bacteriologically confirmed by a second doctor. Once again, the contacts of the 2 cases were subsequently moved into isolated accommodation; in addition, all of the cases' clothing and bedclothes were burnt.

## Results

The following analysis aggregates data from the 3 pneumonic plague outbreaks due to their small sample sizes.

### Symptomatic period

**Figure 2:**
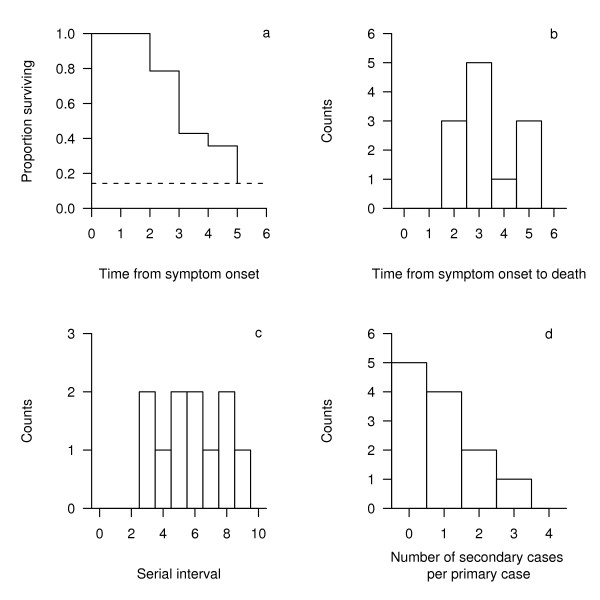
**(a) Kaplan-Meier survival curve; dashed horizontal line represents 1-case fatality ratio, (b) histogram of the symptomatic periods of fatal cases (n = 12), (c) histogram of the time between successive cases (n = 11), (d) histogram of transmission (n = 12)**.

Figure 2a shows the Kaplan-Meier survival function following symptom onset. All cases that died experienced at least 2 days of symptoms and survived for no longer than 3 further days. 2 of the 14 cases survived the disease giving a case fatality ratio of 86% with a 95% binomial confidence interval of {57%, 98%}. Figure 2b shows a histogram of the symptomatic period for the 12 fatal cases giving a mean and standard deviation (SD) of 3.3 and 1.2 days, respectively. A Kolmogorov-Smirnov (KS) test showed evidence against the sample data here being drawn from the log-normal distribution as reported by Gani & Leach [3] who calculated a mean and SD of 2.5 and 1.2 days, respectively (p-value = 0.02).

### Serial interval

The serial interval (symptom onset time in a primary case to symptom onset time in a secondary case) could only be calculated for 11 of the 14 cases since the remaining 3 were index cases whose source of infection was not explicitly identified. The estimated serial intervals ranged from 3 to 9 days with a mean and SD of 5.8 and 2.0 days, respectively (Figure 2c). Nishiura *et al*. have previously reported 2 independent serial interval distributions; the first giving a mean and SD of 5.7 and 3.6 days, respectively,[5] and the second giving equivalent parameters of 5.1 and 2.3 days [6]. A KS test revealed no evidence against the sample data here being drawn from either gamma distribution (first distribution p-value = 0.38, second distribution p-value = 0.22).

### Secondary cases

Figure 2d shows a histogram of the number of secondary cases per primary case in the observed minor outbreaks prior to the implementation of any control measures giving a mean (termed *R*_*minor*_) of 0.9 (SD = 1.0), slightly lower than the *R*_*minor *_of 1.3 (SD = 1.8) reported by Gani & Leach [3]. A visual inspection of the histogram shows a similar shape to the geometric distribution provided by Gani & Leach and confirmed by Lloyd-Smith *et al*.,[17] but the KS test is only valid for testing against continuous distributions and therefore cannot be applied here. Despite this, the geometric distribution was again superior (Akaike's Information Criterion with a correction for small sample sizes (AIC_c_) = 29.5) to either the Poisson (AIC_c _= 32.7) or negative-binomial (AIC_c _= 35.6) models. The results here also compare favourably with the *R*_*minor *_values of 0.9 for Mukden in 1946 and 1.1 for Madagascar in 1957 [3]. Finally, there was insufficient data to provide any statistical comparison with the time-decreasing *R*_*minor *_analysed by Nishiura *et al*.,[6] although it is noteworthy that all 3 index cases here infected only 1 other person.

### Secondary attack rate

Let the household secondary attack rate (SAR) be defined as the number of secondary cases resulting from each household index case divided by the number of household contacts of each index case. The family living in Charity Farm Cottages, Shotley, consisted of about 8 persons [13] giving a household SAR of 14% with a 95% binomial confidence interval of {0%, 58%}. 3 children remained disease-free at Latimer Cottages, Freston, giving a household SAR of 20% with a 95% binomial confidence interval of {1%, 72%}. The early isolation of Mrs P prevented any further cases amongst her husband or their 6 children [13] making the household SAR untenable for Turkey Farm Cottages, Freston. It should be noted that 4 doctors, 3 nurses and 2 church members also had close contact with the Freston cases but none of them developed the disease [13,18]. The lack of information regarding the number of inhabitants at either Brickhill Terrace Cottages, Shotley, or Warren Lane Cottages, Erwarton, means that the household SAR cannot be calculated for either residence.

## Discussion

There seems to be sufficient evidence in the Erwarton outbreak to suggest that public health interventions were implemented too late to prevent any further cases because contacts were isolated at approximately the time of the second death (i.e. after any additional transmission would have occurred). The situation is slightly less clear in Shotley where pneumonic plague was only accepted as the disease responsible many years later - all deaths were registered as being due to acute pneumonia and any explicit isolation was not reported. It is important to note that Dr Carey, who attended cases in all 3 outbreaks, undoubtedly encouraged barriers to close contact which may have implicitly affected the epidemiology of each outbreak. In spite of this, Mr C and Mrs P were still infected by Mrs C during the Freston outbreak even though Dr Carey had impressed on those nursing Mrs C of the necessity of avoiding close contact whenever possible [19]. This highlights the difficulty of quantifying such medical advice from outbreak data - a subject perhaps more appropriately addressed through behavioural research studies [20].

2 of the 3 Suffolk outbreaks were what are usually referred to as 'minor outbreaks' which by definition decline to extinction with or without the strong influence of public health interventions. By analysing the entire transmission tree of a minor outbreak it is natural that one calculates an *R*_*minor *_estimate slightly smaller than 1; this consequence is clear even without any explicit estimation. Nevertheless, it is not appropriate to regard that the average number of secondary cases per primary case in a fully susceptible population (i.e. *R*_0_) of pneumonic plague is less than 1 in general and that pneumonic plague is not capable of causing a major epidemic. For example, when evaluating the major epidemic in Manchuria, 1910,[5] which was clearly dominated by human-to-human transmission (due to confirmation of the absence of bubo amongst the cases), *R*_0 _of pneumonic plague is definitely regarded as greater than 1. What the present study and previous studies [3,6,17] have tended to analyse are examples in which the outbreak declined to extinction before growing to a major epidemic, and thus, the resulting estimate of the average number of secondary cases per single primary case is not a true representation of *R*_0_. This is apparent from branching process theory given that an observation of a single epidemic is merely "a single sample path profile" [21]. Furthermore, the underlying social contact structure that predicates *R*_0 _is unclear in many settings and so interpretation of transmissibility inferences between settings requires care.

The case fatality ratio of pneumonic plague is often stated as approaching 100% and so it is interesting that 14% of the Suffolk cases survived, although the small sample size leads to wide confidence intervals. Of the 14 possible cases of pneumonic plague only 3 were confirmed bacteriologically (Mr C and Mrs P at Freston, and Mrs G at Erwarton). There can be little doubt that the other 2 cases at Latimer Cottages and Mrs B at Warren Lane Cottages also had the disease [9]. However, it is possible that the 2 surviving cases in Shotley did not experience pneumonic plague; indeed, all the cases were originally believed to have been due to a virulent form of influenza [13]. On the other hand, perhaps the strain of *Y. pestis *responsible for the Suffolk outbreaks was less virulent than in other outbreaks resulting in a less than 100% case fatality ratio. It is also possible that the 2 surviving Shotley cases could have initially suffered from bubonic plague before displaying pneumonic symptoms, although no buboes were reported. Interestingly, the presumed bubonic plague outbreak of 1909/1910 in the nearby village of Trimley resulted in 7 cases and 4 deaths - 6 of these cases were described as having a "knot" (enlarged gland) in the neck, axilla or groin [8].

The plague outbreaks that occurred in Suffolk during the early twentieth century did not behave like the 'black death' pandemic of the 14^th ^- 17^th ^centuries (which killed a quarter of the population of Europe) but more like sylvatic plague [9,22]. Enzootic amongst wild rodents in many areas of the world, sylvatic plague (a term that is used to reflect the ecological rather than the medical context of the disease) rarely results in the infection of more than a few individuals or single households. Interestingly, the index cases of all 3 outbreaks here seem to have followed a direct course of primary pneumonic plague (which has also been associated with sylvatic plague [23]) rather than experiencing the usual secondary effects after suffering bubonic symptoms. It should be noted that there was 1 further case that experienced secondary pneumonic plague - on 10^th ^October 1911, a sailor, Mr B, was admitted to the sick quarters of the Royal Naval Barracks at Shotley. Mr B was probably infected 3 days earlier after he cut himself while cleaning a rabbit that he had caught less than a mile from Latimer Cottages, Freston. Soon after developing a severe pneumonia on 15^th ^October, Mr B was isolated after inspection of his sputum suggested plague. No transmission occurred and Mr B finally recovered on 12^th ^January 1912.

The last pandemic of plague started in China, 1894, and spread to many parts of the world including India where over 1 million people were killed by the disease [9]. Plague reached Glasgow in 1900 [24] resulting in 36 bubonic cases and 16 deaths. Prior to this outbreak, Britain remained effectively free from plague for nearly 250 years following the great plague of London (1665-1666) that caused 60,000 deaths in a population of 450,000. The absence of plague was probably due to the introduction of the brown rat (*Rattus norvegicus*) which eventually replaced the common black rat (*Rattus rattus*) [8]. Since the brown rat prefers to live apart from man, as opposed to the black rat which prefers human habitations, the close contact required for flea-based transmission is likely to have decreased over time. However, over 200 species of wild rodents are capable of harbouring plague [8] and could act as a reservoir for potential human infection following an aerosolised release of *Y. pestis*. Indeed, the small localised outbreaks seen in Suffolk could provide a model of potential secondary outbreaks of plague after any initial epidemic has been curtailed, with domesticated cats perhaps providing the most direct rodent-human link in contemporary western society [25,22].

## Conclusions

The average symptomatic period of the cases described here was almost 1 day longer than that found by Gani & Leach [3] in their analysis of a variety of outbreaks, although the 2-5 day range fell within previously reported values. The main difference between the results of these 2 papers is that none of the cases here died within the first day of experiencing symptoms whereas approximately 15% of cases suffered a 1 day infectious period in the Gani & Leach study. The smaller sample size of the Suffolk outbreaks perhaps offers the most likely explanation for this discrepancy; although possible epidemiological differences cannot be ruled out. The average ~6 day serial interval agrees closely with values reported by Nishiura *et al*. [5,6] and in 2 situations where it was possible to estimate, the household SAR was approximately 15%, but again the small sample sizes lead to wide confidence intervals. These outbreaks highlight that non-professional caregivers are particularly vulnerable and would likely comprise the majority or non-index pneumonic plague cases following importation of the disease or deliberate release of the causative organisms. Finally, it should be emphasised that even with *R*_*minor *_= 0.9, significant amplification of any index cases could ensue through human-to-human transmission [3] and would need to be considered appropriately in terms of risk assessment and public health mitigation strategies.

## List of Abbreviations

AIC: Akaike's Information Criterion; KS: Kolmogorov Smirnov; SAR: Secondary Attack Rate; SD: Standard Deviation.

## Competing interests

The author declares that he has no competing interests.

## Authors' contributions

JE analysed the data and wrote the paper.

## Author Information

JE is a Mathematical Modeller for the Health Protection Agency. His interests include the development of mathematical models to assess and predict the potential public health impacts of newly emerging infectious diseases and the likely relative benefits of different mitigation strategies.
